# A flow-level dataset of WireGuard tunnel traffic with matched encrypted-side features and application labels

**DOI:** 10.1016/j.dib.2026.112696

**Published:** 2026-03-18

**Authors:** Yasameen Sajid Razooqi, Adrian Pekar

**Affiliations:** a Department of Networked Systems and Services, Faculty of Electrical Engineering and Informatics, Budapest University of Technology and Economics, Műegyetem rkp. 3., H-1111 Budapest, Hungary; b CUJO LLC, Budapest, Hungary

**Keywords:** NFStream, Network measurement, VPN, NonVPN, Tunnel metadata, Encrypted traffic analysis, Flow feature extraction

## Abstract

This data article describes a flow-level dataset derived from paired captures on both sides of a WireGuard virtual private network tunnel. Pre-tunnel traffic was recorded on the inner tunnel interface before encapsulation, and encrypted transport traffic was recorded on the outer side, using a GL.iNet Flint 2 (GL-MT6000) router, an inline network TAP, and a Linux capture host. Two capture sessions totaling approximately 80 h of residential broadband traffic from 10 devices were recorded with nanosecond-precision packet timestamps; the released flow-level data uses millisecond resolution as exported by NFStream. The raw captures were cleaned to retain TCP and UDP packets and to remove non-initial IPv4 fragments. Flow records were generated from the cleaned inner-side captures using NFStream, which assigned each flow an application name and application category label via deep packet inspection. Inner packets were matched to outer WireGuard transport data packets using time alignment and a padded-length consistency rule, and matched packets were attributed to flows using 5-tuple keys with temporal and capacity constraints. Encrypted-side statistics were then aggregated per flow. The released dataset consists of two Parquet files, one per capture session, that combine NFStream flow fields, including application labels and inner-side per-packet sequences for the first 255 packets, with encrypted-side derived attributes such as matched packet counts, byte totals, durations, rates, direction-specific byte volumes, packet-size statistics, inter-arrival time distributions, size-ratio metrics and outer-side per-packet sequences for the first 255 packets. This cross-correlation structure pairing pre-tunnel application labels with encrypted tunnel-side features, can support research on encrypted traffic classification, application identification, VPN detection, and feature engineering for flow-level analysis under encryption.

Specifications Table

**Table:** 

Subject	Computer Sciences
Specific subject area	Encrypted network traffic analysis and VPN flow-level classification
Type of data	Table (Parquet), Processed
Data collection	Traffic captured using tcpdump on a GL.iNet Flint 2 (GL-MT6000) router (inner WireGuard interface) and a Linux host connected to an inline network TAP (outer encrypted side). Two sessions recorded on a residential broadband connection in Budapest, Hungary, through a Surfshark WireGuard endpoint in Prague, Czech Republic. Nanosecond-precision packet timestamps used during capture and matching. Flows exported using NFStream (millisecond precision) with application labeling via nDPI. Inner packets matched to outer WireGuard transport packets by time alignment and padded-length consistency. Encrypted-side statistics aggregated per flow.
Data source location	Institution: FlowFrontiers Research Group, Department of Networked Systems and Services, Budapest University of Technology and Economics (BME) City: Budapest · Country: Hungary
Data accessibility	Repository name: Zenodo Data identification number: https://doi.org/10.5281/zenodo.18700746 Direct URL to data: https://zenodo.org/records/18700746 Instructions for accessing these data: The main two Parquet files are publicly available in the repository (VPN-nonVPN-Dataset) and can be downloaded directly.
Related research article	*none*

## 1 Value of the Data

• The dataset [1] pairs pre-tunnel application labels with encrypted-side flow features for the same traffic. Each flow carries an application name and category label derived from pre-tunnel deep packet inspection, alongside per-flow statistics computed solely from encrypted tunnel packets. This cross-correlation structure enables supervised training of classifiers that predict application identity or traffic category using only features observable from the encrypted tunnel exterior.
• The encrypted-side features include per-flow packet-size statistics, inter-arrival time distributions for inbound and outbound directions, size ratios between encrypted and pre-tunnel packets, direction-specific byte volumes, and rate measures. Per-packet sequences for the first 255 packets of each flow (direction, size, and inter-arrival time) are also included, enabling early-flow feature extraction without access to raw packet captures. These attributes support feature engineering and model development for encrypted traffic classification at both sub-flow and full-flow granularity.
• Each flow is labeled at two granularity levels: individual application name and broader application category. These dual labeling supports both fine-grained application identification and coarse-grained traffic categorization, allowing researchers to evaluate classification performance at multiple levels of specificity.
• The dataset is released as two processed Parquet files, one per capture session, eliminating the need for PCAP handling, protocol decoding, or custom flow extraction, and enabling direct integration into machine learning pipelines. This format facilitates reproducible experimentation and fair comparison across classification methods and datasets.
• The dataset directly supports several benchmark tasks for encrypted traffic analysis: (1) application classification, where the goal is to predict application_name or application_category_name from the 32 encrypted-side features, evaluated using macro-averaged F1 score to account for class imbalance; (2) early-flow classification, where application identity is predicted using only the first (N) packets from the per- packet sequence arrays (splt_direction, splt_ps, splt_piat_ms) and their outer-view counterparts (outer_splt_direction, outer_splt_ps, outer_splt_piat_ms), with performance reported as a function of packet-prefix length; and (3) cross-session generalization, where a model is trained on one session and evaluated on the other to measure temporal robustness, compared against within-session cross validation as a baseline.
• Existing public VPN traffic datasets vary in labeling methodology, capture scope, and protocol coverage [[2], [3], [4]]. This dataset provides an independently collected flow-level resource for cross-dataset validation and robustness analysis, complementing existing benchmarks for encrypted traffic classification. As the data reflect a single residential network, one VPN protocol (WireGuard), and one provider endpoint, generalization from models trained on this dataset is bounded to comparable edge and home network settings.
• The application category distribution exhibits extreme class imbalance characteristic of natural residential traffic: Web and Network together account for approximately 85% of flows, while categories such as Game, Video, Advertisement, Media, and DataTransfer each contain fewer than 50 flows per session. The raw per-flow labels are provided without aggregation, allowing researchers to define their own grouping strategies, apply oversampling or cost-sensitive learning, or exclude low-count categories depending on the target task.

## 2 Background

Virtual private network tunnels encrypt and encapsulate user traffic, replacing application-layer identifiers with uniform encrypted transport. For network operators, this limits the ability to characterize traffic traversing their infrastructure, creating challenges for regulatory compliance and network management [5]. As VPN adoption grows, research interest in encrypted traffic analysis has expanded, with common directions including VPN detection, application identification, and traffic category classification [[6], [7], [8]]. Recent surveys highlight recurring challenges related to dataset design, labelling, and capture conditions in existing VPN traffic datasets [9].

This dataset was compiled to address the need for a labeled flow-level resource that links pre-tunnel application identifiers to encrypted tunnel-side observations. The underlying traffic was collected on a residential broadband connection through a WireGuard tunnel, with paired captures on both sides enabling deterministic packet matching and per-flow aggregation. The resulting cross-correlation between application labels and encrypted-side features provides ground truth for developing and evaluating classifiers that must operate without payload access.

## 3 Data Description

The Data Description presents the structure and contents of the released dataset. It outlines the organization of the Parquet files, describes the flow-level record schema and included feature groups, and reports summary statistics that characterize traffic across both capture sessions.

### 3.1 Dataset overview

The released dataset consists of two Parquet files, one per capture session, containing flow-level records derived from residential broadband traffic routed through a WireGuard virtual private network tunnel. Each row represents a single bidirectional network flow. The two sessions were recorded in December 2025 and cover approximately 80 h of household traffic from 10 devices. The dataset contains 226,454 flows in total: 122,975 flows in Session 1 and 103,479 flows in Session 2.

Each Parquet file contains 126 columns. Of these, 89 are NFStream-exported flow attributes [10], including the flow identifier, source and destination addresses and ports, transport protocol, timing and volume statistics, TCP flag counts, sequence of first packet lengths and inter-arrival times (SPLT), application name, application category name, and deep packet inspection metadata. Five additional columns store flow assignment metadata (flow identifier, start and end timestamps, forward and reverse 5-tuple keys). The remaining 32 columns are encrypted-side derived features computed from matched WireGuard tunnel packets, (columns 95–126 in Table 2). All timestamps in the released files are in milliseconds, as exported by NFStream; the nanosecond precision used during packet capture and matching is not preserved in the flow-level output.

No fields in the dataset support individual device attribution. As described in the Limitations section, IP masquerading on the WireGuard interface replaces original device addresses with the VPN-assigned tunnel address (10.14.0.2), so all flows share the same local IP endpoint. The MAC address columns (src_mac, dst_mac) and their corresponding OUI fields (src_oui, dst_oui) are uniformly set to 00:00:00:00:00:00 and 00:00:00, respectively, because the WireGuard interface operates at Layer 3 without Ethernet framing. Consequently, neither IP addresses nor MAC addresses can be used for device-level grouping or identification.

### 3.2 Repository structure and files

Table 1 lists the files released in the public data repository. The main dataset is provided as session level Parquet flow records. Intermediate Parquet files capture the packet matching output and the inner side NFStream flow exports used for flow assignment and verification. The processing scripts reproduce the Parquet and CSV outputs from the original PCAP captures. The Validation folder provides notebooks to regenerate reported statistics, a worked example flow record, and SHA-256 checksums for integrity checking. Separate license files specify CC BY 4.0 for data and MIT for code.

**Table 1:** Repository contents and dataset files.

File name	Format	Description
Data/session_1/session1_flows.parquet	Parquet	Session 1 flow records after packet to flow aggregation
Data/session_1/session1_nfstream_inner_flows. parquet	Parquet	Session 1 inner side NFStream flow export
Data/session_1/session1_packet_matches. parquet	Parquet	Session 1 matched inner-outer packet pairs
Data/session_2/session2_flows.parquet	Parquet	Session 2 flow records after packet to flow aggregation
Data/session_2/session2_nfstream_inner_flows. parquet	Parquet	Session 2 inner side NFStream flow export
Data/session_2/session2_packet_matches. parquet	Parquet	Session 2 matched inner-outer packet pairs
Code/packet_matching.py	Python	Packet matching script (Phase 3a)
Code/flow_matching.py	Python	Flow matching and aggregation script (Phase 3b&3c)
Code/compress_parquets.py	Python	Compresses the Parquet files using ZSTD to reduce file size, after converting CSV files to Parquet
LICENSE/Code LICENSE.txt	TXT	MIT License for scripts
LICENSE/Data LICENSE.txt	TXT	CC BY 4.0 License for data
Validation/Dataset_summary_statistics.ipynb	Jupyter notebook	Reproduces dataset summary statistics and manuscript tables from released data
Validation/Matching_coverage.ipynb	Jupyter notebook	Reproduces matching coverage results and figures
Validation/Worked_example_random_row.csv	CSV	Worked example flow record exported as column name value table
Validation/checksums_sha256.txt	TXT	SHA-256 hashes for raw PCAP inputs and released Parquet files

### 3.3 Flow record structure and feature schema

Each row in the Parquet file corresponds to a single bidirectional network flow extracted from the inner (pre-tunnel) capture using NFStream. The base flow attributes include the NFStream flow identifier, the assigned application label, transport protocol, source and destination ports, and NFStream-exported timing and volume statistics such as first and last observation times, packet counts, and byte totals. These fields preserve the original NFStream flow representation and provide the reference timeline for subsequent processing.

Among the NFStream-exported fields, three columns store per-packet sequences for the first 255 packets of each flow: splt_direction records the packet direction (source-to-destination or destination-to-source), splt_ps records the packet size in bytes, and splt_piat_ms records the inter-arrival time in milliseconds. To provide the same sequence-level view on the encrypted side, the derived-feature block includes corresponding outer SPLT columns: outer_splt_direction, outer_splt_ps, and outer_splt_piat_ms. In both representations, unused entries beyond the actual packet count are padded with −1. These per-packet arrays preserve the order and timing of early-flow packets, enabling computation of sub-flow statistics for any prefix of up to 255 packets without access to the original PCAP files. This complements the aggregate flow-level statistics, which summarize measurements over the entire flow duration.

Table 2 provides the complete data dictionary for all 126 columns.

**Table 2:** Complete data dictionary.

#	Column name	Type	Unit	Description	Missing/padding encoding
1	id	int	—	NFStream flow identifier (sequential)	—
2	expiration_id	int	—	Flow expiration reason (0 = idle timeout, 1 = active timeout, 2 = custom, 3 = natural end)	—
3	src_ip	string	—	Source IP address (VPN-assigned 10.14.0.2 or remote)	—
4	src_mac	string	—	Source MAC address	Always 00:00:00:00:00:00 (L3 tunnel)
5	src_oui	string	—	Source OUI (first 3 bytes of MAC)	Always 00:00:00
6	src_port	int	—	Source transport port (TCP or UDP)	—
7	dst_ip	string	—	Destination IP address	—
8	dst_mac	string	—	Destination MAC address	Always 00:00:00:00:00:00 (L3 tunnel)
9	dst_oui	string	—	Destination OUI	Always 00:00:00
10	dst_port	int	—	Destination transport port	—
11	protocol	int	—	Transport protocol number (6 = TCP, 17 = UDP)	—
12	ip_version	int	—	IP version (4 or 6)	—
13	vlan_id	int	—	VLAN identifier	0 if none
14	tunnel_id	int	—	Tunnel identifier	0 if none
15	bidirectional_first_seen_ms	int	ms	Timestamp of first packet in either direction	—
16	bidirectional_last_seen_ms	int	ms	Timestamp of last packet in either direction	—
17	bidirectional_duration_ms	int	ms	Flow duration (last − first)	0 for single-packet flows
18	bidirectional_packets	int	packets	Total packet count in both directions	≥ 1
19	bidirectional_bytes	int	bytes	Total byte count in both directions	≥ 1
20	src2dst_first_seen_ms	int	ms	First packet time, source to destination	—
21	src2dst_last_seen_ms	int	ms	Last packet time, source to destination	—
22	src2dst_duration_ms	int	ms	Duration, source to destination	0 for single-packet direction
23	src2dst_packets	int	packets	Packet count, source to destination	≥ 0
24	src2dst_bytes	int	bytes	Byte count, source to destination	≥ 0
25	dst2src_first_seen_ms	int	ms	First packet time, destination to source	0 if no reverse traffic
26	dst2src_last_seen_ms	int	ms	Last packet time, destination to source	0 if no reverse traffic
27	dst2src_duration_ms	int	ms	Duration, destination to source	0 if no reverse traffic
28	dst2src_packets	int	packets	Packet count, destination to source	0 if unidirectional
29	dst2src_bytes	int	bytes	Byte count, destination to source	0 if unidirectional
30	bidirectional_min_ps	int	bytes	Minimum packet size, bidirectional	—
31	bidirectional_mean_ps	float	bytes	Mean packet size, bidirectional	—
32	bidirectional_stddev_ps	float	bytes	Standard deviation of packet size, bidirectional	0 for single-packet flows
33	bidirectional_max_ps	int	bytes	Maximum packet size, bidirectional	—
34	src2dst_min_ps	int	bytes	Minimum packet size, source to destination	—
35	src2dst_mean_ps	float	bytes	Mean packet size, source to destination	—
36	src2dst_stddev_ps	float	bytes	Std dev of packet size, source to destination	0 for single-packet direction
37	src2dst_max_ps	int	bytes	Maximum packet size, source to destination	—
38	dst2src_min_ps	int	bytes	Minimum packet size, destination to source	0 if no reverse traffic
39	dst2src_mean_ps	float	bytes	Mean packet size, destination to source	0 if no reverse traffic
40	dst2src_stddev_ps	float	bytes	Std dev of packet size, destination to source	0 if no reverse traffic
41	dst2src_max_ps	int	bytes	Maximum packet size, destination to source	0 if no reverse traffic
42	bidirectional_min_piat_ms	int	ms	Minimum packet inter-arrival time, bidirectional	0 for single-packet flows
43	bidirectional_mean_piat_ms	float	ms	Mean packet inter-arrival time, bidirectional	0 for single-packet flows
44	bidirectional_stddev_piat_ms	float	ms	Std dev of inter-arrival time, bidirectional	0 for single-packet flows
45	bidirectional_max_piat_ms	int	ms	Maximum inter-arrival time, bidirectional	0 for single-packet flows
46	src2dst_min_piat_ms	int	ms	Minimum inter-arrival time, source to destination	0 for single-packet direction
47	src2dst_mean_piat_ms	float	ms	Mean inter-arrival time, source to destination	0 for single-packet direction
48	src2dst_stddev_piat_ms	float	ms	Std dev of inter-arrival time, source to destination	0 for single-packet direction
49	src2dst_max_piat_ms	int	ms	Maximum inter-arrival time, source to destination	0 for single-packet direction
50	dst2src_min_piat_ms	int	ms	Minimum inter-arrival time, destination to source	0 if no reverse traffic
51	dst2src_mean_piat_ms	float	ms	Mean inter-arrival time, destination to source	0 if no reverse traffic
52	dst2src_stddev_piat_ms	float	ms	Std dev of inter-arrival time, destination to source	0 if no reverse traffic
53	dst2src_max_piat_ms	int	ms	Maximum inter-arrival time, destination to source	0 if no reverse traffic
54	bidirectional_syn_packets	int	packets	SYN flag count, bidirectional	0 for UDP flows
55	bidirectional_cwr_packets	int	packets	CWR flag count, bidirectional	0 for UDP flows
56	bidirectional_ece_packets	int	packets	ECE flag count, bidirectional	0 for UDP flows
57	bidirectional_urg_packets	int	packets	URG flag count, bidirectional	0 for UDP flows
58	bidirectional_ack_packets	int	packets	ACK flag count, bidirectional	0 for UDP flows
59	bidirectional_psh_packets	int	packets	PSH flag count, bidirectional	0 for UDP flows
60	bidirectional_rst_packets	int	packets	RST flag count, bidirectional	0 for UDP flows
61	bidirectional_fin_packets	int	packets	FIN flag count, bidirectional	0 for UDP flows
62	src2dst_syn_packets	int	packets	SYN flag count, source to destination	0 for UDP flows
63	src2dst_cwr_packets	int	packets	CWR flag count, source to destination	0 for UDP flows
64	src2dst_ece_packets	int	packets	ECE flag count, source to destination	0 for UDP flows
65	src2dst_urg_packets	int	packets	URG flag count, source to destination	0 for UDP flows
66	src2dst_ack_packets	int	packets	ACK flag count, source to destination	0 for UDP flows
67	src2dst_psh_packets	int	packets	PSH flag count, source to destination	0 for UDP flows
68	src2dst_rst_packets	int	packets	RST flag count, source to destination	0 for UDP flows
69	src2dst_fin_packets	int	packets	FIN flag count, source to destination	0 for UDP flows
70	dst2src_syn_packets	int	packets	SYN flag count, destination to source	0 for UDP flows
71	dst2src_cwr_packets	int	packets	CWR flag count, destination to source	0 for UDP flows
72	dst2src_ece_packets	int	packets	ECE flag count, destination to source	0 for UDP flows
73	dst2src_urg_packets	int	packets	URG flag count, destination to source	0 for UDP flows
74	dst2src_ack_packets	int	packets	ACK flag count, destination to source	0 for UDP flows
75	dst2src_psh_packets	int	packets	PSH flag count, destination to source	0 for UDP flows
76	dst2src_rst_packets	int	packets	RST flag count, destination to source	0 for UDP flows
77	dst2src_fin_packets	int	packets	FIN flag count, destination to source	0 for UDP flows
78	splt_direction	string (array)	—	Per-packet direction for first 255 packets (0 = src→dst, 1 = dst→src)	−1 for unused positions beyond actual packet count
79	splt_ps	string (array)	bytes	Per-packet size for first 255 packets	−1 for unused positions
80	splt_piat_ms	string (array)	ms	Per-packet inter-arrival time for first 255 packets; first entry is 0	−1 for unused positions
81	application_name	string	—	nDPI-assigned application name (e.g., DNS, TLS.Facebook, QUIC)	``Unknown'' if unidentified
82	application_category_name	string	—	nDPI-assigned category (e.g., Web, Network, Chat)	``Unspecified'' if unidentified
83	application_is_guessed	int	—	Whether nDPI used its giveup heuristic (1) or classified via standard DPI analysis (0)	—
84	application_confidence	int	—	nDPI confidence level (0 = unknown, 1 = match by port, 3 = DPI partial, 5 = DPI cache, 6 = full DPI, 7 = match by IP)	—
85	requested_server_name	string	—	TLS Server Name Indication or HTTP Host	``0” if not available
86	client_fingerprint	string	—	TLS client fingerprint (JA3-style hash)	``0” if not available
87	server_fingerprint	string	—	TLS server fingerprint (JA3S-style hash)	``0” if not available
88	user_agent	string	—	HTTP User-Agent header value	``0” if not available
89	content_type	string	—	HTTP Content-Type header value	``0” if not available
90	flow_id	int	—	Flow identifier used for packet-to-flow assignment	—
91	flow_start_ms	float	ms	Flow start time used during assignment (= bidirectional_first_seen_ms)	—
92	flow_end_ms	float	ms	Flow end time used during assignment (= bidirectional_last_seen_ms)	—
93	k5_fwd	string	—	Forward 5-tuple key (src_ip|dst_ip|roto|src_port|dst_port)	—
94	k5_rev	string	—	Reverse 5-tuple key (dst_ip|src_ip|proto|dst_port|src_port)	—
95	matched_packets	float	packets	Number of matched packet pairs assigned to this flow	0 if no matches
96	outer_bytes	float	bytes	Sum of outer padded lengths over matched packets	0 if no matches
97	first_matched_time_ms	float	ms	Earliest inner timestamp among assigned packets	0 if no matches
98	last_matched_time_ms	float	ms	Latest inner timestamp among assigned packets	0 if no matches
99	outer_duration_ms	float	ms	Matched duration on inner timeline (last − first)	0 if no matches or single packet
100	outer_first_matched_time_ms	float	ms	Earliest outer timestamp among assigned packets	0 if no matches
101	outer_last_matched_time_ms	float	ms	Latest outer timestamp among assigned packets	0 if no matches
102	outer_capture_duration_ms	float	ms	Matched duration on outer timeline (last − first)	0 if no matches or single packet
103	mean_outer_pkt_size	float	bytes	Mean outer padded length of matched packets	0 if no matches
104	std_outer_pkt_size	float	bytes	Std dev of outer padded length of matched packets	0 if no matches or single packet
105	outer_packet_rate	float	packets/s	Matched packets per second (over outer_duration_ms)	0 if no matches
106	outer_byte_rate	float	bytes/s	Outer bytes per second (over outer_duration_ms)	0 if no matches
107	outer_bytes_in	float	bytes	Sum of outer padded lengths for inbound matched packets	0 if none
108	outer_bytes_out	float	bytes	Sum of outer padded lengths for outbound matched packets	0 if none
109	mean_size_ratio	float	—	Mean of (outer_padded_length / inner_length) per matched packet	0 if no matches
110	std_size_ratio	float	—	Std dev of size ratio	0 if no matches or single packet
111	max_size_ratio	float	—	Maximum size ratio observed	0 if no matches
112	outer_min_piat_ms_in	float	ms	Minimum inter-arrival time, inbound matched packets	0 if fewer than 2 inbound matches
113	outer_mean_piat_ms_in	float	ms	Mean inter-arrival time, inbound matched packets	0 if fewer than 2 inbound matches
114	outer_stddev_piat_ms_in	float	ms	Std dev of inter-arrival time, inbound	0 if fewer than 2 inbound matches
115	outer_max_piat_ms_in	float	ms	Maximum inter-arrival time, inbound	0 if fewer than 2 inbound matches
116	outer_min_piat_ms_out	float	ms	Minimum inter-arrival time, outbound matched packets	0 if fewer than 2 outbound matches
117	outer_mean_piat_ms_out	float	ms	Mean inter-arrival time, outbound matched packets	0 if fewer than 2 outbound matches
118	outer_stddev_piat_ms_out	float	ms	Std dev of inter-arrival time, outbound	0 if fewer than 2 outbound matches
119	outer_max_piat_ms_out	float	ms	Maximum inter-arrival time, outbound	0 if fewer than 2 outbound matches
120	outer_min_piat_ms	float	ms	Minimum inter-arrival time, pooled across directions	0 if fewer than 2 total matches
121	outer_mean_piat_ms	float	ms	Mean inter-arrival time, pooled	0 if fewer than 2 total matches
122	outer_stddev_piat_ms	float	ms	Std dev of inter-arrival time, pooled	0 if fewer than 2 total matches
123	outer_max_piat_ms	float	ms	Maximum inter-arrival time, pooled	0 if fewer than 2 total matches
124	outer_splt_direction	string (array)	—	Per-packet direction for first 255 packets (0 = src→dst, 1 = dst→src)	−1 for unused positions beyond actual packet count
125	outer_splt_ps	string (array)	bytes	Per-packet size for first 255 packets	−1 for unused positions
126	outer_splt_piat_ms	string (array)	ms	Per-packet inter-arrival time for first 255 packets; first entry is 0	−1 for unused positions

### 3.4 Encrypted side to NFStream feature mapping

Table 3 maps each encrypted-side derived column to its nearest NFStream counterpart to support comparison between the inner (pre-tunnel) and encrypted-side views of the same flow. It also clarifies conceptual differences, particularly where encrypted-side metrics are computed from a matched packet subset and rely on padded-length representations rather than plaintext byte counts. This mapping supports feature interpretation and selection in supervised learning settings, where models are trained to infer application or traffic category using only encrypted tunnel-side statistics.

**Table 3:** Encrypted-side columns mapped to nearest NFStream counterparts.

Encrypted-side column	Description	Nearest NFStream counterpart
matched_packets	Matched packet pairs assigned to flow	bidirectional_packets
outer_bytes	Sum of outer padded lengths	bidirectional_bytes
first_matched_time_ms	Earliest inner time of assigned packets	bidirectional_first_seen_ms
last_matched_time_ms	Latest inner time of assigned packets	bidirectional_last_seen_ms
outer_duration_ms	Matched duration on inner timeline	bidirectional_duration_ms
outer_first_matched_time_ms	Earliest outer time of assigned packets	bidirectional_first_seen_ms (inner-side equivalent)
outer_last_matched_time_ms	Latest outer time of assigned packets	bidirectional_last_seen_ms (inner-side equivalent)
outer_capture_duration_ms	Matched duration on outer timeline	bidirectional_duration_ms (outer-side equivalent)
mean_outer_pkt_size	Mean outer padded length	bidirectional_mean_ps
std_outer_pkt_size	Std dev of outer padded length	bidirectional_stddev_ps
outer_packet_rate	Matched packets per second	bidirectional_packets / (bidirectional_duration_ms / 1000)
outer_byte_rate	Outer bytes per second	bidirectional_bytes / (bidirectional_duration_ms / 1000)
outer_bytes_in	Outer padded length sum, inbound	dst2src_bytes
outer_bytes_out	Outer padded length sum, outbound	src2dst_bytes
mean_size_ratio	Mean of outer_padded_length / inner_length	No direct counterpart (cross-view ratio)
std_size_ratio	Std dev of size ratio	No direct counterpart
max_size_ratio	Maximum size ratio	No direct counterpart
outer_min_piat_ms_in	Min inter-arrival time, inbound	dst2src_min_piat_ms
outer_mean_piat_ms_in	Mean inter-arrival time, inbound	dst2src_mean_piat_ms
outer_stddev_piat_ms_in	Std dev of inter-arrival time, inbound	dst2src_stddev_piat_ms
outer_max_piat_ms_in	Max inter-arrival time, inbound	dst2src_max_piat_ms
outer_min_piat_ms_out	Min inter-arrival time, outbound	src2dst_min_piat_ms
outer_mean_piat_ms_out	Mean inter-arrival time, outbound	src2dst_mean_piat_ms
outer_stddev_piat_ms_out	Std dev of inter-arrival time, outbound	src2dst_stddev_piat_ms
outer_max_piat_ms_out	Max inter-arrival time, outbound	src2dst_max_piat_ms
outer_min_piat_ms	Min inter-arrival time, pooled	bidirectional_min_piat_ms
outer_mean_piat_ms	Mean inter-arrival time, pooled	bidirectional_mean_piat_ms
outer_stddev_piat_ms	Std dev of inter-arrival time, pooled	bidirectional_stddev_piat_ms
outer_max_piat_ms	Max inter-arrival time, pooled	bidirectional_max_piat_ms
outer_splt_direction	First 255 outer packet directions	splt_direction
outer_splt_ps	First 255 outer packet sizes (bytes)	splt_ps
outer_splt_piat_ms	First 255 outer packet PIAT values (ms)	splt_piat_ms

### 3.5 Dataset summary statistics

Table 4 summarizes the per-session flow counts, protocol breakdown, and application label diversity.

**Table 4:** Per-session dataset summary.

Metric	Session 1	Session 2
Total flows	122,975	103,479
TCP flows	57,033 (46.4%)	49,014 (47.4%)
UDP flows	65,942 (53.6%)	54,465 (52.6%)
Unique application names	81	75
Unique application categories	20	19
Flows with zero matched packets	19 (0.02%)	14 (0.01%)

Table 5 shows the distribution of flows across application categories as assigned by NFStream. The distribution of application categories is heavily skewed. Web and Network account for approximately 85% of flows in both sessions, while several categories such as Game, Video, Advertisement, Media, DataTransfer, and VPN contain fewer than 50 flows each. These near-empty categories reflect the natural traffic composition of a residential network and are retained as reported by nDPI to preserve labeling fidelity. The dataset does not aggregate or relabel these categories, leaving researchers free to define their own grouping strategies. For supervised classification, practitioners may choose to merge low-count categories into a single minority class, apply oversampling or cost-sensitive learning, or exclude them depending on the target task. The raw per-flow labels are provided to support any of these approaches.

**Table 5:** Flow counts per application category.

Application category	Session 1	Session 2
Web	61,588 (50.1%)	49,102 (47.5%)
Network	45,612 (37.1%)	38,836 (37.5%)
Collaborative	3074 (2.5%)	3558 (3.4%)
Cloud	3205 (2.6%)	2573 (2.5%)
Unspecified	2366 (1.9%)	3314 (3.2%)
SocialNetwork	2406 (2.0%)	1477 (1.4%)
Chat	1891 (1.5%)	2094 (2.0%)
VoIP	824 (0.7%)	777 (0.8%)
System	768 (0.6%)	462 (0.4%)
SoftwareUpdate	360 (0.3%)	377 (0.4%)
Download	283 (0.2%)	368 (0.4%)
Email	219 (0.2%)	172 (0.2%)
Database	127 (0.1%)	200 (0.2%)
ConnCheck	189 (0.2%)	92 (0.1%)
VPN	38 (0.0%)	46 (0.0%)
DataTransfer	11 (0.0%)	8 (0.0%)
Media	7 (0.0%)	17 (0.0%)
Advertisement	5 (0.0%)	5 (0.0%)
Video	1 (0.0%)	1 (0.0%)
Game	1 (0.0%)	—

Table 6 lists the top 15 application names by flow count per session. Application names follow the nDPI naming convention used by NFStream [11], where a prefix such as TLS or HTTP indicates the detected transport protocol, and a suffix indicates the identified service. DNS, TLS, and QUIC account for most flows in both sessions.

**Table 6:** Top 15 application names by flow count.

Application name	Session 1	Session 2
DNS	44,947 (36.5%)	38,594 (37.3%)
TLS	41,755 (34.0%)	33,891 (32.8%)
QUIC	17,222 (14.0%)	11,852 (11.5%)
Unknown	2366 (1.9%)	3314 (3.2%)
TLS.Facebook	2023 (1.6%)	1175 (1.1%)
TLS.MS_OneDrive	1662 (1.4%)	1627 (1.6%)
HTTP	1450 (1.2%)	2655 (2.6%)
TLS.Microsoft365	1320 (1.1%)	1159 (1.1%)
TLS.Teams	1252 (1.0%)	848 (0.8%)
WhatsApp	1148 (0.9%)	655 (0.6%)
NTP	768 (0.6%)	462 (0.4%)
TLS.HuaweiCloud	638 (0.5%)	—
STUN	599 (0.5%)	—
HTTP.UbuntuONE	585 (0.5%)	396 (0.4%)
TLS.Microsoft	542 (0.4%)	437 (0.4%)
Discord	—	1443 (1.4%)
Telegram	—	1038 (1.0%)

Table 7 presents flow duration, flow size, and per-flow packet count distributions for both sessions.

**Table 7:** Flow duration, size, and packet count distribution.

Statistic	Session 1	Session 2
**Flow duration (seconds)**		
Min	0.000	0.000
Median	35.49	12.07
Mean	16,740.7	9506.6
Max	173,570.0	115,078.8
**Flow size (bytes)**		
Min	36	36
Median	5526	2756
Mean	324,515	393,396
Max	514,135,667	1686,905,577
**Packets per flow**		
Min	1	1
Median	21	15
Mean	338	412
Max	736,743	1945,930
**Matched packets per flow**		
Min	0	0
Median	21	15
Mean	338	411
Max	736,521	1945,708

### 3.6 Matching coverage

**Fig. 1:**
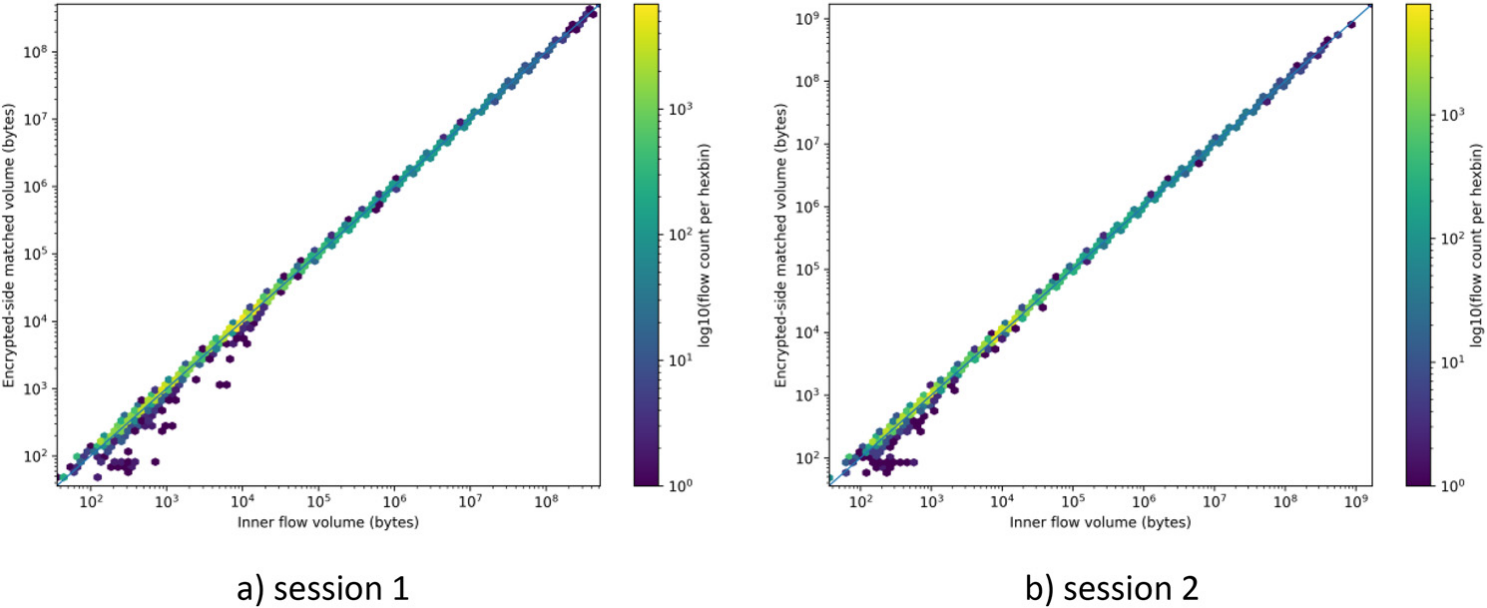
Encrypted-side matched volume versus inner flow volume, Session 1 and 2.

Fig. 1 shows the relationship between inner-side flow volume (as exported by NFStream) and the corresponding encrypted-side matched volume for each flow, across both capture sessions. Each hexagonal bin represents the number of flows at a given coordinate on log-log axes, with color indicating the logarithm of flows per bin. The diagonal reference line marks the *y* = *x* relationship. Most flows align closely with the *y* = *x* diagonal, indicating high volume consistency between inner and encrypted-side observations.

**Fig. 2:**
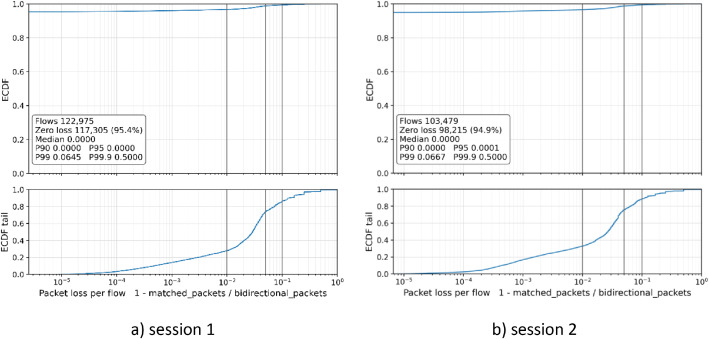
Distribution of packet-level matching loss per flow, Session 1 and 2.

Fig. 2 shows the distribution of per-flow packet matching loss, defined as one minus the ratio of matched packets to the bidirectional packet count reported by NFStream. The upper panels display the full empirical cumulative distribution function. The lower panels show the non-zero loss tail on a logarithmic scale. In both sessions, approximately 95% of flows have zero packet loss. Fewer than 1% of flows exceed 6–7% loss, and the 99.9th percentile is approximately 50%.

## 4 Experimental Design, Materials and Methods

This section describes the experimental setup and data processing workflow used to generate the released dataset. It outlines the measurement environment, capture configuration, software toolchain, and the multi-phase pipeline applied to produce the final flow-level Parquet files from paired inner and outer packet traces.

### 4.1 Measurement setting, topology, and instrumentation

The measurements were conducted on a residential broadband ISP connection in Budapest, Hungary. A WireGuard VPN tunnel was established using a GL.iNet Flint 2 (GL-MT6000) router configured as a client and a Surfshark WireGuard service endpoint located in Prague, Czech Republic, as the remote VPN endpoint. Traffic was monitored at two observation points to capture both pre-tunnel and tunneled views. Pre-tunnel packets were collected on the router’s WireGuard client interface on the inner side of the tunnel. Encrypted transport packets were collected on the outer path as UDP 51820 traffic. The recorded sessions reflect typical household network activity.

**Fig. 3:**
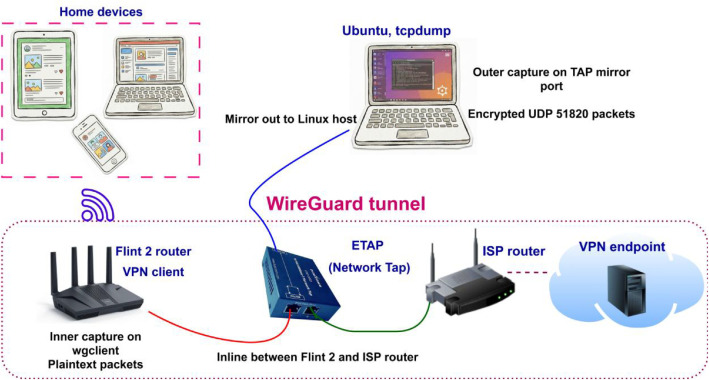
Measurement topology and capture points. Pre-tunnel packets were captured on the Flint 2 (GL-MT6000) router WireGuard client interface. Encrypted tunnel packets were captured on a Linux host connected to an inline network TAP.

Fig. 3 summarizes the topology and instrumentation. The Flint 2 router served as the VPN client gateway for the home devices. A network TAP was placed inline on the link between the Flint 2 and the ISP router and provided a mirrored output to a Linux capture host. This design isolated the outer capture workload from the router and supported stable capture under high packet rates.

### 4.2 Software environment and toolchain

Table 8 lists the software versions used during data collection and processing. The GL.iNet Flint 2 router runs an OpenWrt-based embedded Linux firmware, which provides a standard shell environment and supports tcpdump for interface-level packet capture. Traffic capture and filtering were performed using tcpdump on both the Flint 2 router and the Linux capture host. Field extraction and WireGuard dissection for packet matching were performed using tshark (Wireshark). Flow records were generated from the cleaned inner-side PCAP files using NFStream, a Python framework for network flow metering and flow-level feature extraction. Packet matching and packet-to-flow assignment were implemented in Python using custom scripts included in the data repository.

**Table 8:** Software and environment versions.

Component	Version	Role
Linux capture host	Ubuntu 22.04	Outer-side packet capture and all processing
GL.iNet Flint2 router	OpenWrt 21.02-SNAPSHOT, GL.iNet firmware 4.8.2, kernel 5.4.238	Inner-side packet capture and VPN client gateway
tcpdump	4.99.1	Packet capture and PCAP filtering
Wireshark / tshark	4.6.2	Field extraction and WireGuard protocol dissection
NFStream	6.6.0	Flow metering and application labeling
nDPI	5.0 (bundled with NFStream)	Deep packet inspection for application identification
Python	3.13.5	Packet matching and flow aggregation scripts

### 4.3 Dataset generation workflow

**Fig. 4:**
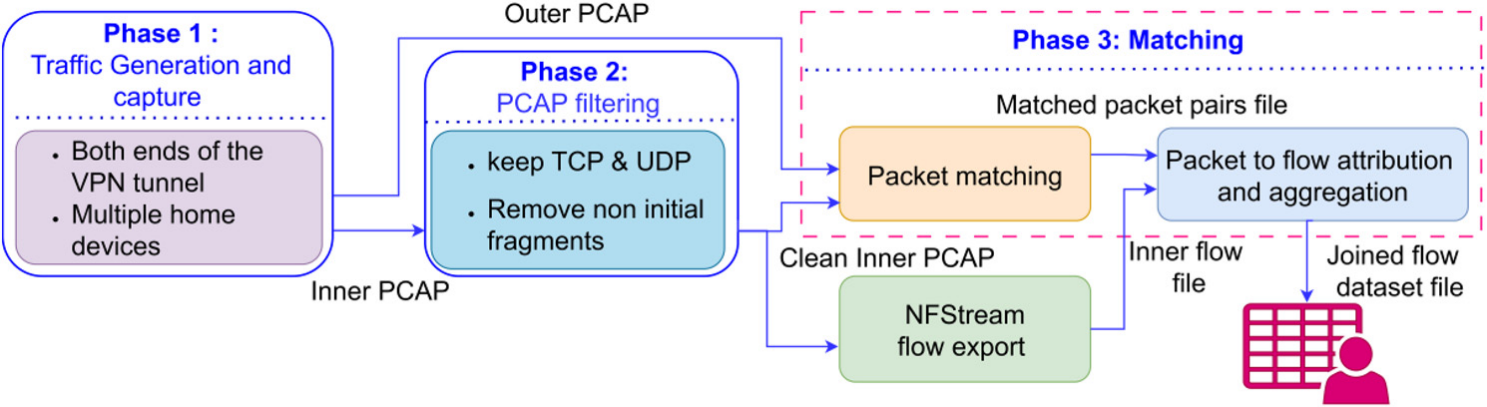
Dataset generation workflow. Phase 1 produces paired inner and outer PCAP files. Phase 2 filters the inner PCAP. Phase 3 matches packets, exports flows, and aggregates encrypted-side statistics into a released Parquet file per session.

The dataset was produced through a three-phase pipeline, as illustrated in Fig. 4. Phase 1 covers traffic generation and capture on both sides of the VPN tunnel. Phase 2 applies filtering to the inner-side PCAP to standardize the packet set for flow export and matching. Phase 3 performs packet matching between the inner and outer captures, exports inner flows using NFStream, assigns matched packets to flows, aggregates encrypted-side statistics per flow, and joins them into a released Parquet file per session. The following subsections describe each phase in detail.

#### 4.3.1 Phase 1: traffic generation and capture

Traffic was generated under normal household use with multiple end-user devices on the home network. Two capture sessions were recorded. Table 9 summarizes the start time, capture duration, inner and outer packet volumes and file sizes, the number of matched packet pairs, and the number of packets assigned to flows.

**Table 9:** Capture session summary. Matched packet pairs show the number of inner-outer matches with the ratio to inner packets in parentheses.

Start date and time	Duration	Inner packets	Inner capture size	Outer packets	Outer capture size	Matched packet pairs	Packets assigned to flows
2025–12–01 17:34:31.75	48 h 16 min 40 s	41,529,447	13 GB	41,554,037	5.84 GB	41,515,695 (0.99)	41,515,695
2025–12–04 11:36:43.87	32 h 0 min 44 s	42,598,408	14 GB	42,620,018	5.99 GB	42,567,568 (0.99)	42,567,568

The capture configuration was selected to support stable recording under high packet rates and to preserve the fields required for later processing, as follows:

• NIC offload features were disabled on both capture points before each capture. With offloading enabled, the network stack and NIC can coalesce and segment packets, producing aggregated frames that do not reflect wire-size packets. On the inner side before encryption, this can yield segments larger than the tunnel MTU (typically 1500 bytes), while on the outer side, encrypted WireGuard packets are already constrained to wire-size. This mismatch would prevent reliable size-based packet matching between the two capture sides. Disabling offloads ensures that captured inner packets reflect actual wire-size transmissions consistent with outer tunnel packets.
• Two traces were recorded for each session:
  1. Inner trace recorded on the Flint 2 router WireGuard client interface. It contains tunnel inner-side packets before encryption.
  2. Outer trace recorded on the Linux interface connected to the TAP mirror. It contains encrypted WireGuard transport traffic observed on UDP port 51820.
• The outer trace used a snapshot length of 128 bytes per packet. This limited stored payload and reduced disk I/O while preserving the required header information for WireGuard transport traffic on UDP port 51820. The inner trace used a larger snapshot length of 384 bytes per packet. This preserved additional header and payload bytes on the tunnel inner side to support application label extraction and flow-level feature generation.
• Nanosecond timestamp precision was enabled on both capture points. Both traces stored nanosecond-resolution packet timestamps generated from the local system clock on each device.
• The output of this phase was four PCAP files, consisting of one inner trace and one outer trace for each of the two capture sessions.

#### 4.3.2 Phase 2: PCAP cleaning

PCAP files were cleaned using tcpdump to standardize the packet set used for flow export and flow enrichment. Only TCP and UDP packets were retained. This kept traffic that carries transport ports and supported stable 5-tuple flow keys. It also aligned the packet set with the flow attribution stage, which relies on port-based identifiers and time windows for deterministic packet-to-flow assignment.

IPv4 non-initial fragments were removed. Non-initial fragments lack transport-layer headers and therefore do not carry source or destination port numbers [12], making it impossible to construct a 5-tuple flow key for these packets. Flow metering tools such as NFStream process only the initial fragment of a fragmented datagram; non-initial fragments are not assigned to flows. Retaining them in the capture would have introduced unmatched packets that could not be attributed to any flow during the packet-to-flow assignment stage. Removing non-initial fragments aligned the inner capture with the packet set that NFStream processes and eliminated a source of systematic mismatch during matching and per-flow aggregation.

The output of this phase was one cleaned inner PCAP per session. The outer PCAPs were not filtered, as they contained only WireGuard UDP transport packets by construction.

#### 4.3.3 Phase 3: matching, flow export, and aggregation

This phase consists of three steps: (1) packet matching between the cleaned inner PCAP and the outer PCAP, (2) flow export from the cleaned inner PCAP using NFStream, and (3) packet-to-flow attribution and aggregation into the final joined Parquet file.

##### 4.3.3.1 Packet matching

Packet matching links each inner-side pre-tunnel packet to a corresponding outer-side WireGuard transport data packet. The procedure produces a packet-level correspondence file that serves as input to the subsequent flow assignment step.

Inner-side packets were represented by frame index, epoch timestamp, IP source and destination, IP protocol, transport ports, and IPv4 total length. When both TCP and UDP port fields were available in the extraction output, TCP ports were used; otherwise UDP ports were used. Outer-side packets were represented by frame index, epoch timestamp, outer IP source and destination, WireGuard counter, and UDP_length_. Outer packets were restricted to WireGuard transport data messages by decoding UDP port 51820 as WireGuard and retaining only type 4 messages. Packets with non-positive derived padded_length_ were excluded, which removes transport keep-alives.

For each outer packet, an estimated padded encrypted payload length was derived by subtracting fixed protocol overheads from the UDP_length_ field (8-byte UDP header, 16-byte WireGuard transport header, and 16-byte authentication tag):

$$\mathrm{padde}d_{\mathrm{leng}\mathrm{th}}=\mathrm{UD}P_{\mathrm{leng}\mathrm{th}}-40$$

For each capture side, the local IP address was inferred from address frequency, with preference for private address ranges. Packets were split into inbound and outbound directions using this local IP. Matching was performed separately per direction and restricted to the overlapping time window between the inner and outer traces. A 15 ms time tolerance was applied during packet matching. Since packets were observed at two distinct capture points, small timing offsets are expected due to transient buffering and processing along the path between the inner tunnel interface and the outer encrypted capture, including WireGuard encapsulation, kernel and driver queueing, and capture-side scheduling. Empirical tuning indicated that a 10 ms tolerance was overly restrictive and increased time-related non-matches, whereas 15 ms improved matching coverage while preserving the padded_length_ consistency constraint. Start and end segments outside the tolerance window were removed to ensure that remaining packets could be aligned under the configured tolerance.

Packets were processed in timestamp order using a single forward scan. For each candidate pair, the time difference and size difference were computed:

$$\mathrm{dt}=\mathrm{oute}r_{\mathrm{time}}-\mathrm{inne}r_{\mathrm{time}}$$

$$\mathrm{pa}d_{\mathrm{diff}}=\mathrm{padde}d_{\mathrm{leng}\mathrm{th}}-\mathrm{inne}r_{\mathrm{leng}\mathrm{th}}$$

A pair was accepted as a match when:

$$\mathrm{abs}\left(\mathrm{dt}\right)\leq \mathrm{tole}\mathrm{rance}\;\;\text{and}\;0\leq \mathrm{pa}d_{\mathrm{diff}}\leq 15$$

The upper bound of 15 bytes on pad_diff_ reflects WireGuard's 16-byte padding rule [13]. WireGuard pads each inner plaintext to a multiple of 16 bytes before encryption, resulting in at most 15 bytes of expansion beyond the original pre-tunnel packet size.

When no match was found, the scan advanced deterministically: the outer index was advanced when the outer packet was ahead in time or too small, and the inner index was advanced when the inner packet was behind in time or too small. When no specific case applied, both indices were advanced.

Matched pairs were streamed to disk as a CSV correspondence file containing the direction label, inner and outer packet fields, dt, pad_diff_, the size ratio, per-direction inter-arrival time and time offset fields, and a canonical 5-tuple key for subsequent flow assignment.

##### 4.3.3.2 Clock synchronization and tolerance sensitivity

Both capture devices synchronized their system clocks via NTP. The Flint 2 router and the Linux capture host each maintained NTP synchronization independently; no additional clock alignment or post-hoc correction was applied. NTP synchronization on a local network typically maintains offsets in the low-millisecond range, well within the 15 ms matching tolerance used for the released dataset.

To quantify the sensitivity of the matching procedure to the choice of time tolerance, the packet matching step was repeated at tolerance values of 5, 10, 15, and 20 ms. Table 10 reports the number of matched packet pairs and the match ratio (matched pairs divided by inner packet count) for each tolerance and session.

**Table 10:** Packet matching sensitivity to time tolerance.

Tolerance (ms)	Session 1 matched pairs	Session 1 match ratio	Session 2 matched pairs	Session 2 match ratio
5	34,904,120	0.84046	39,070,778	0.91718
10	41,131,565	0.99041	42,503,476	0.99777
15	41,515,695	0.99966	42,567,568	0.99927
20	41,512,854	0.99960	42,573,208	0.99940

Match ratios show a clear tolerance effect. At 5 ms, matching drops noticeably (Session 1: 0.840, Session 2: 0.917), indicating that a substantial fraction of true packet pairs has inter-device time offsets between 5 and 10 ms. At 10 ms, match ratios rise sharply (Session 1: 0.990, Session 2: 0.998) and saturate at 15–20 ms (both sessions above 0.999). The slight decrease in Session 1 matched pairs from 15 to 20 ms (41,515,695 to 41,512,854) is an artifact of the greedy forward-scan algorithm, where a wider tolerance can occasionally cause a suboptimal early match that prevents a correct later match; the effect is negligible (0.006%). The 15 ms tolerance used for the released dataset yields near-complete matching with sufficient margin above the observed clock offset range.

##### 4.3.3.3 Flow export and flow assignment

integrates nDPI v5.0 for deep packet inspection (DPI)-based application identification. NFStream passes up to 20 packets per flow (the default `n_dissections` parameter) to nDPI for classification. nDPI was used with default settings; the only non-default option was enabling DNS subclassification, which allows fine-grained DNS labels (e.g., DNS.Google) rather than a generic DNS label. Each flow receives an application name, an application category, a confidence level (0–9, where 6 indicates full DPI-based classification), and a boolean flag (`application_is_guessed`) indicating whether nDPI's giveup heuristic was used when the packet budget was exhausted without a confident identification. Flows that cannot be classified receive the application name ``Unknown'' and category ``Unspecified''. In the released dataset, approximately 85% of flows were classified at confidence level 6 (full DPI), approximately 11–13% were classified via port-based or IP-based matching, and 2–3% remained unclassified.

Matched packets from the correspondence file were assigned to flows using a 5-tuple key (source and destination IP, protocol, source and destination port) in both forward and reverse orientations. Candidate flows were required to share the same key and satisfy temporal consistency, where the packet timestamp falls within the flow time interval. A capacity constraint derived from the NFStream bidirectional packet count prevented over-assignment.

##### 4.3.3.4 Aggregation

After packet-to-flow assignment, encrypted-side measurements were aggregated per flow using outer derived sizes and direction labels. Aggregation computed per-flow volume and rate summaries, distributional statistics for packet sizes and size ratios, and durations based on both the matched inner and outer time ranges. Per-flow inter-arrival time statistics were derived separately for inbound and outbound directions and as a pooled summary. In addition to these aggregate features, outer SPLT sequence columns were extracted per flow (outer_splt_direction, outer_splt_ps, outer_splt_piat_ms) from the first 255 matched outer packets, with −1 padding for shorter sequences. The aggregated summaries and outer SPLT sequences were joined back to the NFStream flow table and exported as a processed Parquet file per session, which together constitute the dataset released with this paper.

### 4.4 Reproduction steps

The following commands reproduce the released Parquet files from the raw inner and outer PCAP captures for one session. The same procedure was applied to both sessions.

**Step 1.** Disable NIC offloads on both capture interfaces before recording:
# Flint 2 router
ethtool -K wgclient gro off gso off tso off lro off rx off tx off
# Linux capture host
sudo ethtool -K enp0s31f6 gro off gso off tso off lro off rx off tx off
**Step 2.** Record inner and outer traces simultaneously:
# Inner capture on Flint 2 (WireGuard client interface, 384-byte snaplen)
tcpdump -i wgclient -s 384 -B 524288 \
–time-stamp-precision nano \
-w /tmp/mountd/disk1_part1/inner_capture.pcap
# Outer capture on Linux (UDP port 51820, 128-byte snaplen)
sudo tcpdump -i〈eth_iface〉 udp port 51820 -s 128 -B 524288 \
–time-stamp-precision nano \
-w /path/outer_capture.pcap
**Step 3.** Filter the inner PCAP to retain only TCP and UDP packets and remove non-initial IPv4 fragments:
tcpdump -r inner_capture.pcap -w filtered_tcp_udp.pcap 'tcp or udp'
tcpdump -r filtered_tcp_udp.pcap -w inner_cleaned.pcap 'not (ip[6:2] & 0 × 1fff != 0)'
**Step 4.** Run packet matching between the cleaned inner and raw outer PCAPs:
python packet_matching.py \
–inner inner_cleaned.pcap \
–outer outer_capture.pcap \
–time-tolerance 15 \
–output packet_matches.csv
**Step 5.** Run flow matching and aggregation to produce the final Parquet file:
python flow_matching.py \
–packets packet_matches.csv \
–flows nfstream_inner_flows.csv \
–output session_flows

## Limitations

All inner-side flows in the released dataset use the WireGuard tunnel IP address (10.14.0.2) as the source or destination endpoint. Capture on the router’s WireGuard interface ensured only tunnel-bound traffic was recorded, but the OpenWrt firewall applies IP masquerading on this interface, replacing original device addresses with the VPN-assigned address. Individual device attribution is not possible, although remaining packet fields were preserved, enabling application-level labeling not available from the encrypted outer side.

The dataset covers only the WireGuard VPN protocol. Other protocols such as OpenVPN or IPsec produce different encapsulation overhead and timing characteristics. A single VPN endpoint provider (Surfshark, Prague) was used; different providers or self-hosted endpoints may introduce different routing and performance characteristics. All traffic was collected on one residential broadband connection in Budapest, Hungary, and network conditions may differ elsewhere.

The encrypted-side size features in the dataset reflect WireGuard's 16-byte padding rule. WireGuard pads each inner plaintext to a multiple of 16 bytes before encrypting with the ChaCha20-Poly1305 stream cipher and appending a fixed 16-byte authentication tag. On the wire, the outer UDP packet therefore includes fixed per-packet overhead (WireGuard transport header, authentication tag, and outer UDP/IP headers) plus 0 to 15 bytes of variable padding. In the released dataset, the encrypted-side size columns (mean_outer_pkt_size, std_outer_pkt_size, size_ratio) are derived from the outer UDP payload length after subtracting the 8-byte UDP header, the 16-byte WireGuard transport header, and the 16-byte authentication tag. The resulting value corresponds to the inner plaintext length plus the 0–15 byte padding residual; outer IP and UDP headers are not included. These features therefore exhibit a protocol-specific 16-byte size granularity. In particular, the size_ratio columns (mean_size_ratio, std_size_ratio, max_size_ratio) and the outer_splt_ps sequence are directly shaped by this padding rule, as the outer padded lengths they record always fall within 0–15 bytes of the corresponding inner plaintext lengths. Classifiers trained on these features may learn patterns specific to WireGuard's 16-byte granularity that do not transfer to protocols with different padding schemes, header sizes, or encapsulation formats. Researchers using this dataset for cross-protocol evaluation should account for this protocol-specific dependency in their feature sets.

Two capture sessions totalling approximately 80 h were recorded; temporal variability across longer periods is not represented. The traffic reflects household usage from 10 devices, which limits applicability to enterprise or backbone settings but is representative of edge and home network scenarios.

Inner and outer timestamps were generated by independent local system clocks, each synchronized via NTP. No dedicated clock drift measurement was performed between the two devices; residual timing offsets are bounded by the matching tolerance and quantified in the sensitivity analysis presented in Section 5.

## Ethics Statement

The authors confirm that they have read and follow the ethical requirements for publication in Data in Brief and confirm that the current work does not involve human subjects, animal experiments, or any data collected from social media platforms. The dataset contains only aggregated flow-level records from a privately controlled residential network; no packet payloads or device-level identifiers are included.

## CRediT Author Statement

**Yasameen Sajid Razooqi:** Methodology, Software, Formal analysis, Investigation, Data curation, Visualization, Writing – Original Draft; **Adrian Pekar:** Conceptualization, Methodology, Resources, Software, Formal analysis, Supervision, Writing – Review & Editing.

## Data Availability

ZenodoVPN-nonVPN-Dataset (Original data).
